# Knowledge and Awareness of Congenital Inguinal and Umbilical Hernia and Its Complications in the Pediatric Population Among Parents in Qassim Region, Saudi Arabia

**DOI:** 10.7759/cureus.71100

**Published:** 2024-10-08

**Authors:** Bandar S Assakran, Norah H Alhumaidi, Ghaday S Almutairi, Faisal S Alkhalifah, Fatimah M Alayed

**Affiliations:** 1 General Surgery, King Fahad Specialist Hospital, Buraydah, SAU; 2 Unaizah College of Medicine, Qassim University, Unaizah, SAU; 3 College of Medicine, Sulaiman Al-Rajhi University, Al-Bukayriyah, SAU; 4 College of Medicine, Qassim University, Buraydah, SAU

**Keywords:** awareness, inguinal hernia, knowledge, pediatric, umbilical hernia

## Abstract

Background

Congenital umbilical and inguinal hernias are prevalent in infants and children. The exact incidence of congenital umbilical hernias remains unclear due to their typically asymptomatic nature, resulting in limited medical attention-seeking by parents. As these hernias are associated with serious complications such as incarceration, strangulation, and, rarely, obstruction, timely recognition and early medical intervention play a critical role in preventing these complications. This study aimed to investigate the level of knowledge and awareness of congenital inguinal and umbilical hernia and its complications in the pediatric population among parents in the Qassim region of Saudi Arabia.

Methodology

This cross-sectional study was conducted among parents living in the Qassim region of Saudi Arabia. A self-administered questionnaire was sent to the targeted parents using an online survey. The questionnaire included sociodemographic data (i.e., gender, marital status, education), general awareness about umbilical and inguinal hernia, and a six-item questionnaire to measure parents’ knowledge regarding umbilical and inguinal hernia.

Results

A total of 440 parents responded to our survey (30.9% males vs. 69.1% females). Overall, 49.5% had at least four or more children. Of the study participants, 19.8% knew of both umbilical and inguinal hernias. The overall median knowledge score of six knowledge items was 2.00 (interquartile range = 2.00). Most parents (64.3%) had poor knowledge, and only 2.3% of them had good knowledge. Higher education, awareness of the meaning of congenital inguinal or umbilical hernias, and having children with a previous history of umbilical or inguinal hernia symptoms were the factors associated with increased knowledge.

Conclusions

The parents’ knowledge regarding congenital umbilical or inguinal hernia was deficient. Parents with self-knowledge of the disease were likely to demonstrate better umbilical and inguinal hernia knowledge than the rest of the groups. This study highlights the importance of awareness campaigns in bridging the knowledge gap.

## Introduction

Congenital umbilical and inguinal hernias are prevalent health issues in infants and children [1]. An umbilical hernia is defined as abdominal protrusion through the umbilical ring, leading to a bulge around the navel [1,2]. The primary etiology of this type of hernia lies in the weakness or incomplete closure of the umbilical ring during fetal development [1]. The exact incidence of congenital umbilical hernias remains unclear due to their typically asymptomatic nature, resulting in limited medical attention-seeking by parents [1-3]. Usually, this type of hernia resolves spontaneously during the first five years of life [4]. However, certain factors such as hernia size, incarceration, or strangulation may complicate these hernias, necessitating immediate surgical intervention [1,2,4].

Inguinal hernias are subdivided into direct and indirect. Direct inguinal hernias are uncommon in infants and children and occur when organs herniate due to weakness in the posterior wall of the inguinal canal [5,6]. On the other hand, most congenital inguinal hernias are indirect. These hernias occur when organs protrude through the inguinal canal due to various fetal developmental factors, such as the persistence of an open inguinal canal [5,6]. The estimated incidence of congenital inguinal hernias among full-term infants ranges from 1% to 5%, with a propensity for right-sided hernias [5,6]. This particular type of hernias is highly associated with serious complications such as incarceration and strangulation, which makes surgical repair the primary treatment for symptomatic or complicated cases and is generally safe and effective [1,5-8].

Multiple studies have been conducted in Saudi Arabia to assess the knowledge about hernias in adults [9-12]. However, the data available regarding parental knowledge about congenital umbilical and inguinal hernias in the pediatric population is limited. Only one study focused on this issue was conducted in Madinah, Saudi Arabia. Among the 474 participants, 46.8% exhibited weak knowledge, while 44.1% had an average knowledge. Interestingly, no significant correlations were observed between knowledge levels and demographic factors such as gender, marital status, education level, or number of children [13].

Detecting congenital umbilical and inguinal hernias early and providing prompt medical intervention is crucial for achieving the best outcomes in infants and children [2]. With this in mind, our study seeks to assess parental awareness of these hernias and their complications in pediatric cases in the Qassim region of Saudi Arabia. By evaluating knowledge levels and awareness among parents, we aim to highlight the importance of recognizing and addressing these conditions promptly for the well-being of children.

## Materials and methods

Study design, sample, and setting

This observational, cross-sectional study was conducted between May 2024 and August 2024 in the Qassim region of Saudi Arabia. The study utilized a convenience sampling technique, which involved administering an online self-administered questionnaire to all accessible subjects who met the inclusion criteria through social platforms.

Sample size

The sample size was estimated to be 384 individuals using the following formula: $(S= Z2*P*(1-P)/M2)$, which was then adjusted using the following formula: $(S/1+[(S-1)/Population])$, where S is the sample size for infinite population, Z is Z score (1.960), P is the population proportion (assumed to be 0.5), and M is the margin of error (assumed to be 0.05). A total of 440 respondents participated in this study.

Inclusion and exclusion criteria

In this study, we included parents living in Qassim, Saudi Arabia who were willing to participate in this study. We excluded parents who did not live in Qassim, Saudi Arabia.

Data collection methods

An online self-administered questionnaire was created via Google Forms and distributed among the participants through social platforms. The questionnaire was developed and validated by the authors of a previous study [13]. The questionnaire was divided into two sections. The first section collected respondents’ demographic data, including gender, residency, marital status, educational level, and the number of children. The second section collected respondents’ general awareness about umbilical and inguinal hernia, as well as a six-item questionnaire to measure parents’ knowledge regarding umbilical and inguinal hernia. The agreement to fill out the self-administered questionnaire was considered consent to participate in the study.

Questionnaire

The parents’ knowledge regarding congenital inguinal and umbilical hernia was assessed using a six-item questionnaire, with the correct answer for each question identified and coded with 1 while the incorrect answer was coded with 0. The total knowledge score was calculated by adding all six items. Scores ranging from 0 to 6 points were achieved. The higher the score, the higher the knowledge about congenital inguinal and umbilical hernia. Parents were classified as having poor knowledge if the score was 2 points or below, scores of 3 to 4 points were classified as moderate, and scores of 5 to 6 points were classified as good knowledge levels.

Statistical analysis

Descriptive statistics were presented as numbers and percentages (%) for all categorical variables, while continuous variables were summarized as mean and standard deviation. The differences in the knowledge score concerning the sociodemographic characteristics and general awareness about congenital inguinal and umbilical hernia were measured using the Mann-Whitney Z test. The normality test was performed using the Shapiro-Wilk test as well as the Kolmogorov-Smirnov test. According to the results, the knowledge score followed the non-normal distribution. Thus, the non-parametric test was applied. Values were considered significant at a p-value of less than 0.05. The data were analyzed using SPSS version 26 (IBM Corp., Armonk, NY USA).

Ethical considerations

Ethical approval was obtained from the Qassim Research Ethics Committee (approval number: 60746427). Participant confidentiality and data protection were ensured throughout the study.

## Results

A total of 440 participants responded to our survey. As shown in Table 1, the majority were mothers (69.1%). Most respondents were married (88%), with nearly half (49.5%) having four or more children. Additionally, 62.9% were bachelor degree holders or higher in education.

**Table 1:** Sociodemographic characteristics of the parents. n = 440.

Study data	N (%)
Gender	
Male	136 (30.9%)
Female	304 (69.1%)
Marital status	
Married	387 (88.0%)
Divorced	27 (06.1%)
Widowed	26 (05.9%)
Number of children	
None	18 (05.9%)
One	70 (15.9%)
Two	63 (14.3%)
Three	71 (16.1%)
Four or more	218 (49.5%)
Educational level	
Middle school	26 (05.9%)
Secondary school	47 (10.7%)
Diploma holder	90 (20.5%)
Bachelor’s degree	230 (52.3%)
Master’s degree	31 (07.0%)
PhD	16 (03.6%)

The general awareness of congenital inguinal or umbilical hernias is presented in Table 2. Notably, 36.8% of respondents claimed that they were aware of umbilical hernia. When asked if one of their children experienced symptoms between 1 month and 12 years old, 13% indicated swelling in the umbilical area. When asked if there was a family history of congenital umbilical or inguinal hernias between 1 month and 12 years of age, 14.1% indicated only umbilical hernia (Table 2).

**Table 2:** General awareness about congenital inguinal or congenital umbilical hernia. n = 440.

Statement	N (%)
Do you know what congenital inguinal or congenital umbilical is?	
Umbilical hernia only	162 (36.8%)
Inguinal hernia only	22 (05.0%)
Both umbilical and inguinal hernias	87 (19.8%)
None of the above	169 (38.4%)
Have you noticed any of your children exhibiting any of the following symptoms when they were between 1 month and 12 years old?	
Swelling in the umbilical area	57 (13.0%)
Swelling in the inguinal area	26 (05.9%)
Both	18 (04.1%)
None of the above	339 (77.0%)
Are there any of your family members who suffered from congenital umbilical hernia and/or inguinal hernias when he/she was between 1 month and 12 years old?	
Umbilical hernia	62 (14.1%)
Inguinal hernia	29 (06.6%)
Both	18 (04.1%)
None of the above	331 (75.2%)

Regarding the assessment of the knowledge of congenital inguinal or umbilical hernias, more than one-third (34.1%) understood the correct meaning of congenital hernia, while those who understood the meaning of congenital inguinal hernia were less (19.8%). Nearly half (42.3%) were aware that congenital umbilical and inguinal hernias can recur even after treatment. Respondents who knew that a part of the intestine was the most common content found inside the bulge of the inguinal hernia or umbilical hernia were 40.5% and 13.9%, respectively. In addition, 52.3% believed an existing relationship exists between undescended testicles in children and inguinal hernia. Based on the above knowledge items, the overall median score of respondents’ knowledge was 2.00 (interquartile range (IQR) = 2.00), with poor, moderate, and good knowledge constituting 64.3%, 33.4%, and 2.3%, respectively (Table 3).

**Table 3:** Assessment of knowledge toward congenital inguinal or congenital umbilical hernia. n = 440. *: Indicates correct answer. Parents were classified as having poor knowledge if the score was 2 points or below, 3 to 4 points were classified as moderate, and scores of 5 to 6 points were classified as good knowledge levels. Total knowledge score (median) = 2.00 (interquartile range = 2.00).

Knowledge item	N (%)
In most uncomplicated cases of umbilical hernia, what is the usual outcome?	
It is a mild condition and does not require a visit to the doctor	36 (08.2%)
It is a mild condition but requires a visit to the doctor*	150 (34.1%)
It is a moderate condition and requires a visit to the doctor	169 (38.4%)
This is a serious condition that requires emergency medical intervention	85 (19.3%)
In most uncomplicated cases of inguinal hernia, what is the usual outcome?	
It is a mild condition and does not require a visit to the doctor	26 (05.9%)
It is a mild condition but requires a visit to the doctor*	87 (19.8%)
It is a moderate condition and requires a visit to the doctor	194 (44.1%)
This is a serious condition that requires emergency medical intervention	133 (30.2%)
Do you think that congenital umbilical and inguinal hernias can recur after treatment?	
Yes, umbilical hernia only	61 (13.9%)
Yes, inguinal hernia only	25 (05.7%)
Yes, both umbilical and inguinal hernias*	186 (42.3%)
No	168 (38.2%)
In your opinion, what is the most common content found inside the bulge of the inguinal hernia?	
Part of the intestine*	178 (40.5%)
Ovaries (in females)	13 (03.0%)
Testicle (in males)	09 (04.3%)
Air	19 (04.3%)
Liquids	47 (10.7%)
I don’t know	174 (39.5%)
In your opinion, what is the most common content found inside the bulge of the umbilical hernia?	
Part of the intestine*	61 (13.9%)
Ovaries (in females)	09 (02.0%)
Testicle (in males)	65 (14.8%)
Air	21 (04.8%)
Liquids	48 (10.9%)
I don’t know	236 (53.6%)
Do you think there is a relationship between undescended testicles in children and inguinal hernia?	
Yes*	230 (52.3%)
No	210 (47.7%)
Level of knowledge	
Poor	283 (64.3%)
Moderate	147 (33.4%)
Good	10 (02.3%)

Measuring the differences in knowledge according to the sociodemographic characteristics and general awareness about congenital inguinal or umbilical hernias, a higher knowledge score was more associated with better education (Z = 2.058; p = 0.040), knowing the meaning of congenital inguinal or congenital umbilical hernia (Z = 7.749; p < 0.001), and a previous history of children exhibiting symptoms of umbilical or inguinal hernia (Z = 2.530; p = 0.011). No significant differences were observed between the knowledge score in terms of gender, marital status, number of children, and family history of congenital umbilical or inguinal hernia (p > 0.05) (Table 4).

**Table 4:** Differences in knowledge score in relation to the sociodemographic characteristics and general awareness about congenital inguinal or congenital umbilical hernia. §: P-value has been calculated using Mann Whitney Z test; **: Significant at p < 0.05 level. n = 440.

Factor	Knowledge score (6), mean ± SD	Z test	P-value ^§^
Gender			
Male	2.19 ± 1.44	1.603	0.109
Female	1.95 ± 1.21		
Marital status			
Married	2.04 ± 1.30	0.299	0.765
Divorced or widowed	1.96 ± 1.18		
Having children			
Yes	1.50 ± 0.99	1.779	0.075
No	2.05 ± 1.29		
Educational level			
Diploma or below	1.85 ± 1.22	2.058	0.040**
Bachelor or higher	2.13 ± 1.32		
Previous history of children exhibiting symptoms of umbilical or inguinal hernia when they were between 1 month and 12 years old			
Yes	2.31 ± 1.15	2.530	0.011**
No	1.94 ± 1.32		
Family history of congenital umbilical hernia and/or inguinal hernias when he/she was between 1 month and 12 years old			
Yes	2.11 ± 1.11	0.932	0.351
No	2.00 ± 1.34		

## Discussion

This study explores parents’ knowledge of congenital inguinal and umbilical hernias and its complications. Local and regional studies show that this subject has not been well documented. Across Saudi Arabia, only one study published parents’ understanding of this disease [13]. Other studies focused more on the general population’s knowledge of adult hernia and its types [9-12] but not specifically on the pediatric population. Thus, this study provides evidence of parents’ understanding of this condition.

The knowledge of parents regarding congenital inguinal and umbilical hernias was inadequate. Nearly two-thirds of our respondents had poor knowledge of the disease, and only 2.3% had good knowledge levels (mean score of 2.02 out of 6 points). Consistent with our findings, a study conducted among parents in the Madinah region found that the knowledge about congenital umbilical and inguinal hernias in infants and children and their complications was weak (46.8%), and the rest were average (44.1%). Without considering pediatric cases, several studies documented a lack of knowledge about adult hernias among the general population [9-11,14-16]. However, this was contradicted by the study by Mafouz et al. (2020) [12], Alsaigh et al. (2023) [17], and Al Judia et al. (2018) [18], which indicates adequate hernia knowledge in the adult population. It is necessary to increase parents’ knowledge of this congenital disease. Health authorities should develop an awareness program targeting parents across the community. Lack of awareness could result in undiagnosed disease and, more likely, complications leading to morbidity and mortality.

Data from this study suggest that education was the only demographic factor associated with congenital hernia knowledge, wherein parents with higher education (bachelor’s degree or higher) demonstrated significantly better knowledge of congenital inguinal or congenital umbilical hernias than parents with lower education (diploma or below). Consistent with this finding, various studies reported an association between hernia knowledge and educational level [11,12,17]. However, our study found no significant association between congenital hernia knowledge concerning gender, marital status, and number of children (p > 0.05). This contradicted the reports of Bahareth et al. (2023) [10]. The study suggested the level of knowledge varied significantly by age, marital status, and occupation. Supporting these reports, Almarhabi et al. (2022) documented that being younger, unmarried, and students were associated with good knowledge levels [16]. In contrast, the findings reported by Helal et al. (2022) found no significant associations between knowledge in terms of age, gender, marital status, level of education, and number of children [13].

The analysis of six knowledge items yielded poor results. Most notably, a lack of understanding of the condition was seen in the basic definition of congenital umbilical hernia and inguinal hernias and when to visit a doctor. Their knowledge of the most frequent content found inside the bulge of inguinal hernia and umbilical hernia had suboptimal results. Only parents’ knowledge of the relationship between undescended testicles in children and inguinal hernia yielded better results. This almost mirrored the report of Helal et al. (2022), where approximately 40% of parents knew congenital umbilical and inguinal hernia is a mild condition requiring a doctor visit [13]. Corroborating these reports, nearly half of the adult population in the western region believed that a hernia was a rupture in the abdomen muscles. Their understanding of the most common risk factors for hernia was limited as well, with more than 85% not being able to recognize smoking, prostate hyperplasia, steroid use, or significant weight loss as hernia-triggering factors in adults [10]. However, in the Aljouf region, the general population exhibited better awareness of the hernia risk factors, including heavy lifting (89.5%), previous surgery (86%), constipation (81%), asthma (59%), enlarged prostate (41%), and smoking (40%) [18].

Parents’ general awareness of umbilical and inguinal hernia also achieved unfavorable results. Findings revealed that although over one-third were aware of what congenital umbilical hernia is, their understanding of inguinal hernia was suboptimal (5%). Further, parents with children who previously experienced hernia symptoms demonstrated better knowledge of the disease (p = 0.011). This supports the results of Alsaigh et al. (2023), with participants who already had hernia showed significantly higher knowledge levels [17].

Limitations

This study has some limitations. First, we did not collect the participants’ age; hence, age comparison of knowledge was not measured. Second, the data on the demographic characteristics are not equally distributed across the groups; hence, the comparison may not be conclusive and needs further investigation. Third, this survey used an online platform to fill out the questionnaire. Hence, some participants may not be truthful with their answers to questions. Fourth, a study of its kind needs to be replicated in different regions to ensure the generalizability of the study findings. Finally, being a cross-sectional study, it is prone to bias and does not measure cause and effect.

## Conclusions

The lack of knowledge about umbilical and inguinal hernia was evident in this study. However, parents with basic information about the disease and who had children who previously experienced symptoms were associated with better knowledge of umbilical and inguinal hernias. Parents’ level of understanding about congenital inguinal and umbilical hernia needs improvement. Health education is key to improving the population’s knowledge of the disease. Education should focus more on participants who were less educated. In addition, social media may play a significant role in disseminating the basic facts of congenital inguinal and umbilical hernias among the population.
